# Bmp Signaling Regulates Hand1 in a Dose-Dependent Manner during Heart Development

**DOI:** 10.3390/ijms22189835

**Published:** 2021-09-11

**Authors:** Mingjie Zheng, Shannon Erhardt, Di Ai, Jun Wang

**Affiliations:** 1 Department of Pediatrics, McGovern Medical School, The University of Texas Health Science Center at Houston, Houston, TX 77030, USA; mingjie.zheng@uth.tmc.edu (M.Z.); shannon.erhardt@uth.tmc.edu (S.E.); 2 The University of Texas MD Anderson Cancer Center UTHealth Graduate School of Biomedical Sciences, The University of Texas Health Science Center at Houston, Houston, TX 77030, USA; 3 Department of Pathology and Laboratory Medicine, School of Medicine, Emory University, Atlanta, GA 30322, USA; di.ai@emory.edu

**Keywords:** Bmp signaling, *Hand1*, Smad, transcriptional regulation, heart development, cardiomyocyte differentiation

## Abstract

The bone morphogenetic protein (Bmp) signaling pathway and the basic helix–loop–helix (bHLH) transcription factor Hand1 are known key regulators of cardiac development. In this study, we investigated the Bmp signaling regulation of *Hand1* during cardiac outflow tract (OFT) development. In *Bmp2* and *Bmp4*loss-of-function embryos with varying levels of *Bmp* in the heart, *Hand1* is sensitively decreased in response to the dose of *Bmp* expression. In contrast, *Hand1* in the heart is dramatically increased in *Bmp4* gain-of-function embryos. We further identified and characterized the Bmp/Smad regulatory elements in *Hand1*. Combined transfection assays and chromatin immunoprecipitation (ChIP) experiments indicated that *Hand1* is directly activated and bound by Smads. In addition, we found that upon the treatment of Bmp2 and Bmp4, P19 cells induced Hand1 expression and favored cardiac differentiation. Together, our data indicated that the Bmp signaling pathway directly regulates *Hand1* expression in a dose-dependent manner during heart development.

## 1. Introduction

Congenital heart defects (CHDs) are the most common birth defects, with an estimated prevalence of 1% in newborns [1]. Cardiac outflow tract (OFT) defects are the most common CHDs and account for one-third of all reported CHDs in human births. The development of the OFT involves interactions and coordination between two types of progenitor pools: second heart field (SHF) progenitors and cardiac neural crest cells (NCCs), regulated by a complex, fine-tuned molecular regulatory network [2,3].

Bone morphogenetic proteins (Bmps) [4] are a family of growth factors belonging to the transforming growth factor beta (TGF-β) superfamily [5]. In the canonical Bmp pathway, Bmp ligands such as Bmp2 and Bmp4 bind to their dual-specificity kinase and heterodimeric receptor complex, consisting of type I and type II receptors, which phosphorylates downstream receptor-regulated Smads (R-Smads), i.e., Smad1, Smad5, and Smad8 (Smad1/5/8) [6,7]. The phospho-R-Smads then form an oligomeric complex with Smad4 and translocate into the nucleus to regulate the expression of downstream genes. The Smad complex can act as both a transcriptional activator and a repressor to regulate target gene expression. The highly conserved Bmp signaling pathway is essential for heart development [8,9], including OFT formation [10,11,12]. Mouse models with Bmp signaling disruptions result in embryonic lethality and CHDs [13,14,15,16,17], as evidenced by *Bmp2*/*4* deletions in the SHF resulting in lethality by embryonic day (E) 12.5 with deficient OFT myocardial differentiation in mice [17].

The hand (the heart- and neural crest derivatives-expressed protein 1) proteins are a subclass of basic helix–loop–helix (bHLH) transcription factors (TFs) that can form homo- and heterodimer combinations with multiple bHLH partners, mediating transcriptional activity in the nucleus [18,19]. Previous studies have shown that Hand1 and Hand2 are core TFs that are expressed in the precardiogenic mesoderm to govern essential gene regulatory networks for cardiovascular growth and morphogenesis [20,21,22,23,24]. *Hand1* plays a vital role in the specification and/or differentiation of extraembryonic structures such as the yolk sac, placenta, and cells of the trophoblast lineages, including cardiac muscle the heart, gut, and sympathetic neuronal development, while also aiding in the proper development of tissues populated by *Hand1*-expressing NCCs [25,26]. *Hand1* deletion in mice results in lethality at E8-8.5, with perturbed heart development at the looping stage [20,27]. Several cardiac studies demonstrated that *Hand1* is an important regulator for cardiac precursor cell fate decision and cardiac morphogenesis [20,21,28,29]. Moreover, mutated *HAND1* has been shown to hinder the effect of *GATA4*, and is associated with congenital heart disease in human patients [30,31]. Mice lacking *Hand2* are embryonically lethal at E10.5, persisting with right ventricular hypoplasia and vascular malformations [22,32]. *HAND2* loss-of-function mutation was found to contribute to human CHDs, and enhanced susceptibility to familial ventricular septal defect (VSD) and double outlet right ventricle (DORV) [33].

In this study, we combined both in vivo mouse genetics and in vitro molecular analyses to investigate the regulation of *Hand1* by Bmp signaling. We found that canonical Bmp-Smad signaling regulates the expression of *Hand1* in a dosage-dependent manner during embryonic heart development, and functions through both cell-autonomous and non-cell-autonomous regulation. Our results suggested that Smads directly bind to the 5′UTR of *Hand1* and activate its expression. In addition, we found that Bmp treatment can activate Hand1 expression and promote the expression of cardiac TFs such as *Nkx2.5* and *Gata4* in P19 cells. Taken together, our data uncovered a fine-tuned canonical Bmp signaling-*Hand1* regulation during heart development.

## 2. Results

### 2.1. Hand1 Expression Decreases in a Dose-Sensitive Manner in Response to Bmp2 and Bmp4 Deficiency during Heart Development

**Figure 1:**
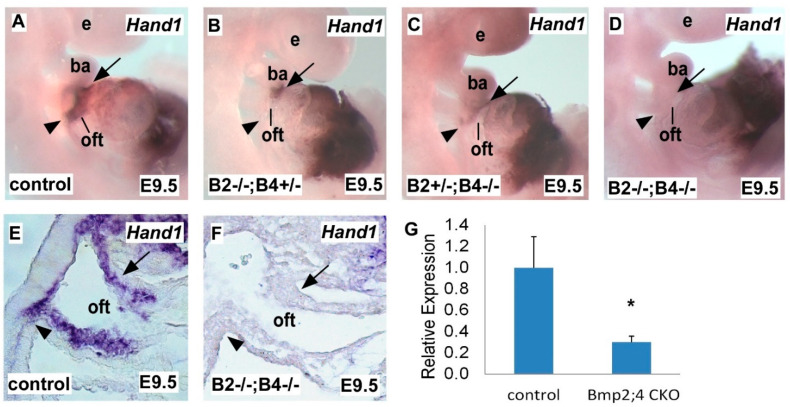
*Hand1* expression is regulated by Bmp signaling in a dose-dependent manner during mouse heart development. (**A**–**D**) Whole-mount in situ hybridization showing *Hand1* expression in mouse embryos at E9.5. (**E**,**F**) E9.5 sagittal sections showed *Hand1* in situ hybridization in the branchial arch and OFT. (**G**) qRT-PCR analysis of *Hand1* expression in control and *Bmp2*/*4* CKO embryos. All genotypes are shown as labeled. *B2−*/*−; B4+*/*−*: *Bmp2* homozygous, *Bmp4* heterozygous mutants; *B2+*/*−; B4−*/*−*: *Bmp4* homozygous, *Bmp2* heterozygous mutants; *B2−*/*−; B4-*/*−*: *Bmp2*/*4* dCKO. e, eye; ba, branchial arch; oft, outflow tract. Arrows and arrow heads point out in situ hybridization signals in OFT and SHF. Data are presented as means ± s.e.m. * indicates *p*-value < 0.05.

SHF progenitors contribute greatly in the formation of the OFT, inflow tract and right ventricle (RV) [34]. To determine *Hand1* expression changes in response to *Bmp* loss-of-function during embryonic OFT development, we generated compound *Bmp2* and *Bmp4* double-conditional knockout (*Bmp2*/*4* dCKO) mutants by crossing the SHF-specific *Mef2c^cre^* driver with the *Bmp2* and *Bmp4* conditional null alleles. Through this cross, we obtained *Bmp2*/*4* dCKO mutants and *Bmp* compound mutants with varying levels of *Bmp* deficiency, including *Bmp2* homozygous, *Bmp4* heterozygous mutants (*Bmp2−*/*−; Bmp4+*/*−*) and *Bmp4* homozygous, *Bmp2* heterozygous mutants (*Bmp2+*/*−; Bmp4−*/*−*). Whole-mount in situ hybridization indicated that compared to the control embryos (Figure 1A), both *Bmp2−*/*−; Bmp4+*/*−* (Figure 1B) and *Bmp2+*/*−; Bmp4−*/*−* (Figure 1C) mutant embryos had a dramatic decrease in *Hand1* expression in the OFT. Strikingly, *Bmp2*/*4* dCKO mutants (Figure 1D) presented with fully abolished *Hand1* expression in the OFT; however, in all *Bmp* mutant samples (compound and dCKO mutants), *Hand1* was still highly expressed in the non-SHF-derived structures such as the left ventricle (LV). These findings indicate that *Hand1* expression is highly sensitive to a Bmp dose-dependent regulation. Histological section analysis of in situ hybridization further confirmed that *Hand1* expression was fully abolished in the OFT of *Bmp2*/*4* dCKO mutant hearts (Figure 1F), as compared to the control hearts with a high expression of *Hand1* in the OFT (Figure 1E). The qRT-PCR analysis further indicated that *Hand1* expression in the hearts of *Bmp2*/*4* dCKO mutants was significantly reduced to around 30% of that of the control hearts at E9.5 (Figure 1G).

During early cardiac development, proliferating SHF progenitor cells add to the OFT and inflow tract, leading to heart tube elongation and its subsequent asymmetric looping formation by E9.5. The other major progenitor cell population contributing to OFT formation is the cardiac NCCs, a highly migratory, multipotent cell population originating from the cranial/vagal region of the dorsal neural tube that subsequently migrates to the OFT. During cardiac morphogenesis, SHF progenitor cells and cardiac NCCs closely interact with each other and coordinately regulate OFT formation [35]. We evaluated expression of the SHF marker *Hand2* and the NCC marker *Ap2* using whole-mount in situ hybridization, and found that the *Bmp2*/*4* dCKO mutant heart had *Hand2* (Figure S1B) and *Ap2* (Figure S1D) expression comparable to that of the control embryo (Figure S1A,C). These findings suggest intact SHF and NCC contributions to the OFT, indicating that the abolished *Hand1* expression in the OFT of the *Bmp2*/*4* dCKO mutant is not caused by reduced cell populations. Importantly, other than in the SHF-derived OFT, *Bmp2*/*4* deletion in the SHF also caused diminished *Hand1* expression in the NCC-derived components of the OFT, suggesting a non-cell-autonomous regulation by *Bmp2*/*4*. Together, these data suggested that during development, Bmp signaling regulates *Hand1* expression in the SHF- and NCC-derived OFT through cell-autonomous and non-cell-autonomous regulation, in a dose-dependent manner.

### 2.2. Hand1 is Upregulated in Bmp4 OE Embryos

**Figure 2:**
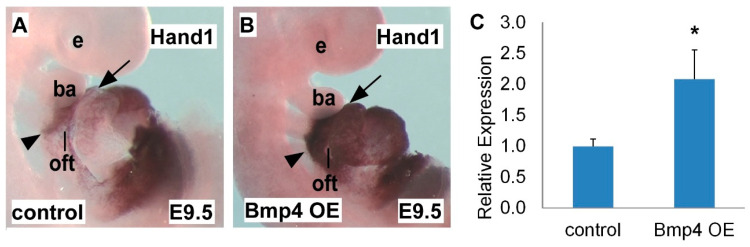
*Hand1* is upregulated upon *Bmp4* overexpression (OE). (**A**,**B**) Whole-mount in situ hybridization of E9.5 *Bmp4* OE embryo compared with control embryo; *Bmp4* OE embryos expanded *Hand1* expression in the SHF and SHF-derived OFT and RV. (**C**) qRT-PCR indicated increased *Hand1* expression level in *Bmp4* OE embryos compared with control embryos. e, eye; ba, branchial arch; oft, outflow tract; rv, right ventricle. Arrows and arrow heads point out in situ hybridization signals in the OFT and SHF. Data are presented as means ± s.e.m. * indicates *p*-value < 0.05.

Finding that *Hand1* expression is sensitive to *Bmp* loss-of-function, we next detected *Hand1* expression in the heart with elevated Bmp signaling. Using a conditional *Bmp4^tetO^* gain-of-function allele (tetracycline inducible) crossed with the *Mef2c^cre^* driver [17,36], we specifically overexpressed *Bmp4* in the SHF-derived heart structures (*Bmp4 OE*). We found that compared with the control heart (Figure 2A), the *Bmp4 OE* mutant heart had robustly expanded *Hand1* expression in the SHF region and SHF-derived structures, including the OFT and RV (Figure 2B), indicated by in situ hybridization staining using a *Hand1* probe. The qRT-PCR results further indicated that the elevated *Bmp4* expression resulted in a significant increase in *Hand1* in the *Bmp4 OE* mutant heart compared with the control heart at E9.5 (Figure 2C). These results indicated that Bmp signaling activates *Hand1* expression during heart development, further supporting the conclusion that *Hand1* expression sensitively responds to Bmp signaling dosage.

### 2.3. Hand1 Is a Direct Target Activated by the Canonical Bmp/Smad Signaling

**Figure 3:**
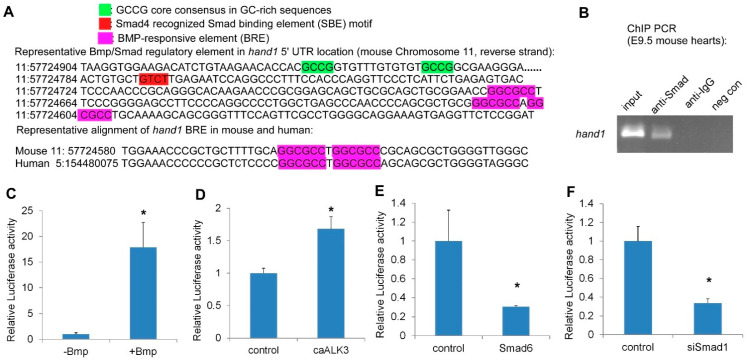
*Hand1* is a direct target activated by the canonical Bmp/Smad signaling. (**A**) Representative Bmp/Smad regulatory element in *Hand1* 5′ UTR location (at upper) and sequence alignment (at lower), showing the conservation among mouse and human (source: Ensembl). (**B**) *In Vivo* ChIP PCR using E9.5 hearts with indicated antibodies to IP chromatin fragment. PCR band (size: 215bp) contains the *Hand*1 5′UTR Bmp/Smad regulatory element. (**C**–**F**) *Hand1* 5′UTR reporter luciferase assays: treated with Bmp (**C**), co-transfected with constitutively active ALK3 (caALK3) (**D**), pcDNA3.1-*Smad6* (**E**), and pSR si*Smad1* (**F**). Data are presented as means ± s.e.m. * indicates *p*-value < 0.05.

Smad TFs function as the major signal transducers for receptors of the Bmp signaling pathway and can interact with specific DNA motifs to regulate gene expression [37,38,39,40]. The R-Smads and Smad4 are composed of two evolutionally conserved domains named Mad Homology 1 and 2 (MH1 and MH2). The MH1 domain is responsible for the Smad binding element’s (SBE) DNA-binding activity, while the MH2 domain is important for heterooligomeric Smad complexes formation and transcriptional activation [41,42]. In addition, based on chromatin immunoprecipitation and structural analysis, Smads have been shown to favor recognizing GC-rich elements (also termed BMP response element (BRE) in certain BMP-responsive genes) [43,44] and CAGAC motifs (also termed Smad binding element (SBE)) [45,46]. To determine if the Bmp/Smad signaling directly regulates Hand1, we undertook sequencing analysis and found that several phylogenetically conserved Smad recognition elements, including the GC-rich elements BRE and SBE, were located in the 5′UTR of *Hand1* (Figure 3A and Figure S2).

To determine whether Smads directly bind to *Hand1*, we performed chromatin immunoprecipitation (ChIP) using a Smad1/5/8 antibody in E9.5 wild-type embryonic heart extracts. There was an obvious enrichment in the anti-Smad1/5/8 immunoprecipitated chromatin compared to the controls, indicating that Smad1/5/8 directly bound to the *Hand1* chromatin (Figure 3B). To evaluate whether the potential Bmp/Smad regulatory elements in *Hand1* are functional, we made a *Hand1* 5′UTR (*Hand1* reporter) luciferase (Luc) reporter and performed luciferase assays in P19 cells. We found that Bmp treatment resulted in a dramatic and significant induction of *Hand1* reporter activity (Figure 3C). Overexpression of the constitutively active Bmpr1a (caALK3) [47] also significantly increased *Hand1* reporter activity (Figure 3D). In contrast, overexpression of *Smad6*, an inhibitory Smad, specifically competed with Smad4 for binding to Smad1 [48], and significantly repressed *Hand1* reporter activity (Figure 3E). *Hand1* Luc reporter activity was also dramatically decreased when using a knockdown *Smad1* short hairpin RNA (shRNA) (Figure 3F). Together, these findings supported the idea that *Hand1* is a direct target activated by the canonical Bmp/Smad signaling.

### 2.4. Bmp Induces Hand1 Expression during Cardiomyogenesis in P19 Cells

**Figure 4:**
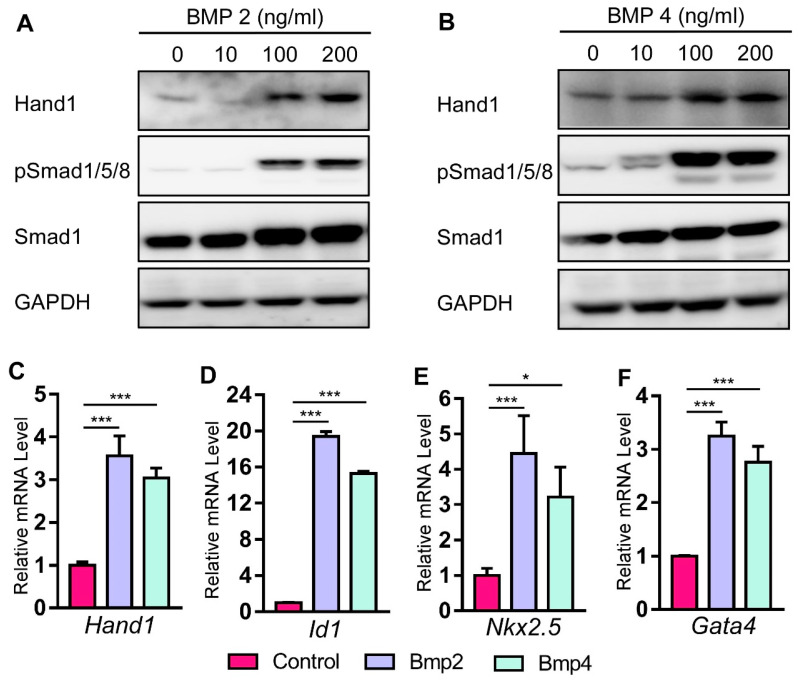
Bmp2 and Bmp4 induce Hand1 expression in P19 cells. (**A**,**B**) P19 cells were stimulated with Bmp2 and 4 for 6 days. Levels of Hand1 were analyzed by western blotting. (**C**–**F**) Total RNA was harvested on day 6 with Bmp treatment for 6 days (100 ng/mL). qRT-PCR was performed for the analysis of *Hand1*, *Id1*, *Nkx2.5*, and *Gata4* mRNA. Data are presented as means ± s.e.m. * indicates *p*-value < 0.05, *** indicates *p*-value < 0.001.

Both in vivo and in vitro studies have established the essential roles of Bmp signals in promoting cardiomyocyte differentiation [49,50,51]. P19 cells are undifferentiated stem cells derived from murine teratocarcinoma [52], which can differentiate into multiple cell types [53,54,55]. Previous studies have indicated that P19 cells can undergo cardiomyogenesis after treatment with chemical inducers such as DMSO, cardiac TFs such as *Mef2c*, and various cytokines [56,57,58,59,60]. It has been shown that Bmp treatment can promote cardiomyocyte differentiation in P19 cells by regulating Nkx2.5 activity [60]. To study *Hand1* expression induced by Bmp2 and Bmp4 during cardiomyogenesis, we treated P19 cells with different concentrations of Bmp2 and Bmp4 for 6 days. Our western blot data indicated that both Bmp2 and Bmp4 induced Hand1 protein expression in a dose-dependent manner (Figure 4A,B). The qRT-PCR analysis also indicated that *Hand1* expression was elevated in P19 cells after 6 days of Bmp2 and Bmp4 treatment (Figure 4C). Transcription factor Id1 is a known direct target of the canonical Bmp/Smad signaling pathway [61,62,63]. Bmp2/4 stimulation also induced *Id1* gene expression, demonstrating that Bmp2 and Bmp4 activate the canonical Bmp/Smad signaling pathway (Figure 4D). Furthermore, we detected an elevated expression of cardiac TFs *Nkx2.5* and *Gata4* with Bmp treatment, indicating undergoing cardiomyogenesis in P19 cells (Figure 4E,F). Taken together, these data showed that the canonical Bmp/Smad signaling pathway induced Hand1 expression during cardiomyogenesis in P19 cells.

## 3. Discussion

In this study, we demonstrated that *Hand1* is a direct downstream target of the canonical Bmp/Smad signaling pathway during heart development. Studies have indicated the importance of *Hand1* and *Hand2* during cardiac morphogenesis, including their contribution in NCCs, myocardium, endocardium, and epicardium. *Hand1* and *Hand2* display different restricted expression patterns in the developing heart. In mice, *Hand1* is highly enriched in the OFT, the cardiomyocytes of the LV, and in the myocardial cuff, between E9.5–13.5 [64]. In contrast, *Hand2* is expressed throughout the linear heart tube, including the RV, the atria, and the left ventricular chambers [65]. Here, we found that the *Hand1* expression level is tightly regulated by Bmp signaling in a dose-dependent manner in the OFT, whereas the *Hand2* expression level is not obviously affected by Bmp signaling activity changes. The *Bmp2−*/*−*; *Bmp4+*/*−* and *Bmp2+*/*−*; *Bmp4−*/*−* compound mutant embryos had low levels of *Hand1* expression in the OFT, which indicated a functional redundancy between *Bmp2* and *Bmp4*. The fully abolished *Hand1* expression in the OFT of the *Bmp2*/*4* dCKO mutant heart indicated that Bmp deletion in the SHF not only regulated *Hand1* expression in the SHF-derived cells, but also *Hand1* expression in neural crest-derived cells, suggesting that Bmp signaling functions in both cell-autonomous and non-cell-autonomous ways. Indeed, Bmp receptors also play essential roles during heart development, such as the Bmp receptor ALK3, that when specifically knocked-out in cardiac myocytes, resulted in cardiac septation and atrioventricular cushion morphogenesis [66]. However, the potential signaling cross talk between SHF progenitors and NCCs in the OFT, mediated by Bmp receptors, will need further investigation. In contrast to *Bmp* loss-of-function, *Bmp* gain-of-function leads to a robust increase in *Hand1* expression, indicating that Bmp signaling is both necessary and sufficient to activate *Hand1* transcription, further supporting the idea that *Hand1* expression sensitively responds to Bmp signaling dosage.

In a facial skeletal development study, Claudio et al. reported that Bmp4 balances self-renewal and differentiation signals in cranial NCCs, and found that compared to the controls, *Hand1* expression was expanded in the developing mandibles of mice with *Bmp4* overexpression in NCCs at E11.5 [36]. In addition, Vincentz et al. found that during mandibular development, Bmp signaling and Hand2 synergistically activate *Hand1* expression, whereas this regulation is inhibited by the homeodomain proteins distal-less homeobox 5 (Dlx5) and Dlx6. However, the Bmp/Hand2 co-regulation and Dlx5/6 antagonism regulation on *Hand1* only occurred in cranial NCCs, not in cardiac NCCs [67]. Here, we found that Bmp signaling in the SHF likely regulates *Hand1* expression in both SHF progenitors and cardiac NCCs during OFT development. However, Hand2 in the SHF likely does not participate in this regulation given that *Hand2* expression was not altered upon Bmp deletion in the SHF.

To further understand the mechanism underlying sensitive expression responses of *Hand1* to Bmp dosages, we analyzed the 5′UTR of *Hand1* and identified conserved Bmp/Smad regulatory elements in the *Hand1* 5′UTR. We made the *Hand1* 5′UTR luciferase reporters and performed a luciferase assay. We found that both the Bmp treatment and overexpression of the constitutively active Bmp receptor (caALK3) induced Hand1 luciferase activity. To further consolidate this result, we also used inhibitory *Smad6* and *Smad1* shRNA to specifically block the Bmp/Smad signaling. We found *Hand1* luciferase reporter activity was decreased when co-transfected with *Smad6* and *Smad1* shRNA. Notably, our ChIP assays’ data showed that Smad1/5/8 binds directly to *Hand1* 5′UTRs in the E9.5 wild-type mouse hearts. These data together indicated that the Bmp regulation on *Hand1* functions through the Smads-mediated canonical Bmp signaling pathway.

Both in vivo and in vitro studies of cardiac cardiomyocyte differentiation systems give strong evidence that Bmps can specifically regulate cardiac differentiation and cardiomyogenesis [59,60,68,69,70]. Our previous work reported that Bmp signaling enhances myocardial differentiation during OFT development [17]. During embryogenesis, *Hand1* is important for the morphogenic patterning and maturation of cardiomyocytes [20,27,29]. The conditional deletion of *Hand1* in cardiomyocytes, using *Nkx2.5^Cre^* or a-myosin heavy chain Cre (*aMHC^Cre^*) driver, results in multiple morphological anomalies that include cardiac conduction system defects, survivable interventricular septal defects, and abnormal LV papillary muscles [29]. Monzen et al. reported that Bmps induce P19 cells for cardiomyocyte differentiation through the mitogen-activated protein kinase kinase kinase TAK1 and cardiac TFs Csx/Nkx-2.5 and GATA-4 [59]. In our in vitro experiments examining *Hand1* expression in P19 cells with treatments of varying Bmp2 and 4 concentrations, we found that both Bmp2 and 4 promote *Hand1* expression in a dose-dependent manner. In addition, after Bmp2 and 4 treatment, cardiac TFs *Nkx2.5* and *Gata4* were also induced when *Hand1* expression was increased. These data, together with previously published findings, suggest that Bmps could potentially activate *Hand1* to promote cardiomyocyte differentiation. However, further electro-physiological experiments in P19 cells and in vivo investigations are still needed in the future.

In conclusion, to our knowledge, this study is the first to demonstrate that the canonical Bmp/Smad signaling pathway in the SHF directly activates *Hand1* expression in a dose-dependent manner during OFT development. Our findings also revealed a potential cell-autonomous and non-cell-autonomous function of Bmp signaling in the SHF and provided better insights into the molecular regulation of OFT development.

## 4. Materials and Methods

### 4.1. Mouse Alleles and Transgenic Lines

The *Bmp2* and *Bmp4* conditional null, *Bmp4^tetO^* gain-of-function allele and the *Mef2c^cre^* line were previously described [17,36].

### 4.2. Antibodies and Reagents

Antibodies used in this study include P-Smad1/5/8 (Cell Signaling Technology, #13820, Danvers, MA, USA), Smad1 (Upstate Biotechnology, Lake Placid, NY, USA), Hand1 (R&D systems, AF3168-SP, Minneapolis, MN, USA), and GAPDH antibody (Abcam, #ab9485, Cambridge, UK). Bmp2 (R&D, #355BM, Minneapolis, MN, USA) and Bmp4 (R&D, #314BP, Minneapolis, MN, USA) proteins were purchased from R&D systems.

### 4.3. Whole-Mount In Situ Hybridization

Whole-mount and section in situ hybridization was performed as previously described [17]. The plasmids for *Hand1* and *Hand2* in situ probes were previously described [71]. For all the experiments, at least three controls and mutant embryos were analyzed for each probe.

### 4.4. Quantitative Real Time RT-PCR

Total RNA from embryonic hearts was isolated using the RNeasy Micro Kit (QIAGEN) [17]. Total RNA from P19 cells was extracted using TRIzol reagent (Life technologies, Carlsbad, CA, USA) following the manufacturer’s protocol. For qRT-PCR assays, iScript Reverse Transcription Supermix (Bio-Rad, Hercules, CA, USA) was used for RT-PCR, and SYBR Green PCR Master Mix (Applied Biosystems, Waltham, MA, USA) was used for real-time thermal cycling (Applied Biosystems, Waltham, MA, USA). All error bars represent SEM. Primers used for qRT-PCR were Gapdh forward, 5′-TGGCAAAGTGGAGATTGTTGCC-3′. Gapdh reverse, 5′-AAGATGGTGATGGGCTTCCCG-3′. *Hand1* forward, 5′-GCCTACTTGATGGACGTGCT-3′. *Hand1* reverse, 5′-CAACTCCCTTTTCCGCTTGC-3′. Gata4 forward, 5′-CCCTGGAAGACACCCCAATC-3′. Gata4 reverse, 5′-TTTGAATCCCCTCCTTCCGC-3′. Nkx2.5 forward, 5′-TGCTCTCCTGCTTTCCCAGCC-3′. Nkx2.5 reverse, 5′-CTTTGTCCAGCTCCACTGCCTT-3′. Id1 forward, 5′-TTGGTCTGTCGGAGCAAAGCGT-3′. Id1 reverse, 5′-CGTGAGTAGCAGCCGTTCATGT-3′.

### 4.5. Chromatin Immunoprecipitation

E9.5 wild-type mice embryonic hearts were dissected and followed by chromatin immunoprecipitation (ChIP) analysis, which was performed using a ChIP assay kit (Upstate) [17]. The two primers for amplifying the Bmp/Smad regulatory element in the 5′ upstream of the *Hand1* genomic sequence were sense, 5′-AACCCGCAGGGCACAAGAA-3′, and antisense, 5′-TGGTTGTGCAAGAGATTGTGA-3′. The PCR product was evaluated for appropriate size on a 2% agarose gel and was confirmed by sequencing. As negative controls, no antibody was used; in addition, normal rabbit immunoglobulin G was used as a replacement for the anti-Smad1/5/8 (sc-6031-R, Santa Cruz) to reveal nonspecific immunoprecipitation of the chromatin.

### 4.6. Luciferase Reporter Assays

Expression and reporter plasmids were described above. Constitutively active ALK3 (caALK3), pcDNA3.1-*Smad6* expression plasmid, and pSR siSmad1 plasmid were previously described [17]. To generate the *Hand1* luciferase reporter plasmid, 2314bp 5′upstream of *Hand1* genomic sequence was amplified using a high-fidelity PCR system (Roche) with two oligonucleotides, sense, 5′-ACGCGTAGGGTACAAAGGGAAACTGGGTGT-3′ (underlined letters indicate the MluI restriction site introduced for subcloning), and antisense, 5′-CTCGAGTGCTCACTCCCTGTACTGAACCTA-3′ (underlined letters indicate the XhoI restriction site introduced for subcloning), and subcloned into pGL3-Basic vector (Promega). P19 cells were transfected using Lipofectamine 3000 (Invitrogen). Luciferase activity assays were performed using the Luciferase Assay System (Promega).

### 4.7. Western Blotting

Western blot was performed as previously described using standard techniques [72]. After 6 days with or without Bmp2/4 treatment, P19 cells were harvested and lysated using 0.5% NP-40 lysis buffer (50 mM Tris-HCl pH 7.5, 150 mM NaCl, 0.5% NP-40, 10% glycerol, phosphatase and protease inhibitors) for 10 min on ice, and centrifuged at 14,000 rpm for 10 min at 4 °C. For Western blot analysis, the proteins were loaded and separated by SDS-PAGE, and transferred onto a PVDF membrane (Millipore, IPVH00010). The membranes were blocked in 5% non-fat milk for 1 h at room temperature and incubated with primary antibodies overnight at 4 °C. The membranes were incubated with HRP-conjugated secondary antibodies for 2 h at room temperature and were imaged by Bio-rad imaging systems. Antibodies used for immunoblotting are mentioned above.

### 4.8. Cell Culture

Mouse embryonic carcinoma cell line P19 were maintained in Minimum Essential Medium (MEM) supplemented with 10% fetal bovine serum (FBS) at 37 °C in a humidified incubator with 5% CO_2_. P19 cells were seeded at a concentration of 0.5 × 10^6^ cells per well in 6-well plates and cultured for 24 h to reach 100% confluence (day 0). To induce differentiation, cells were washed in PBS and cultured in MEM supplemented with 10% fetal bovine serum (FBS), Bmp2 or Bmp4, referred to as differentiation medium.

## Data Availability

Data are contained within the article or Supplementary Materials.

## Supplementary Materials

The following are available online at https://www.mdpi.com/article/10.3390/ijms22189835/s1, Figure S1: *Hand2* is not changed in control and *Bmp2/4* mutant; Figure S2: Diagram of the Bmp/Smad regulatory element in the 5′ upstream of *H**and**1* genomic sequence (at upper) and its phylogenetic sequence alignment (at lower).
