# Supplementary material for: Bmp Signaling Regulates Hand1 in a Dose-Dependent Manner during Heart Development
Source: Int J Mol Sci. 2021 Sep 11;22(18):9835. doi: 10.3390/ijms22189835 (PMC8465227; doi:10.3390/ijms22189835)
Supplement: Supplementary file 1 [file ijms-22-09835-s001.zip › ijms-1344577-supplementary.pdf]

Supplementary information

Bmp signaling regulates Hand1 in a dose-dependent manner during heart development

Mingjie Zheng [1], Shannon Erhardt [1,2], Di Ai[3] and Jun Wang [1,2,*]

1 Department of Pediatrics, McGovern Medical School, The University of Texas Health Science Center at Houston, Houston, TX, 77030, United States; mingjie.zheng@uth.tmc.edu (M.Z.), shannon.erhardt@uth.tmc.edu (S.E.), jun.wang@uth.tmc.edu (J.W.)

2 Graduate School of Biomedical Sciences, The University of Texas MD Anderson Cancer Center and The University of Texas Health Science Center at Houston, Houston, TX, 77030, United States; shannon.erhardt@uth.tmc.edu (S.E.), jun.wang@uth.tmc.edu (J.W.)

3 Department of Pathology and Laboratory Medicine, Emory University, Atlanta, GA, 30322, United States; di.ai@emory.edu (D.A.)

*Correspondence: Jun Wang; jun.wang@uth.tmc.edu

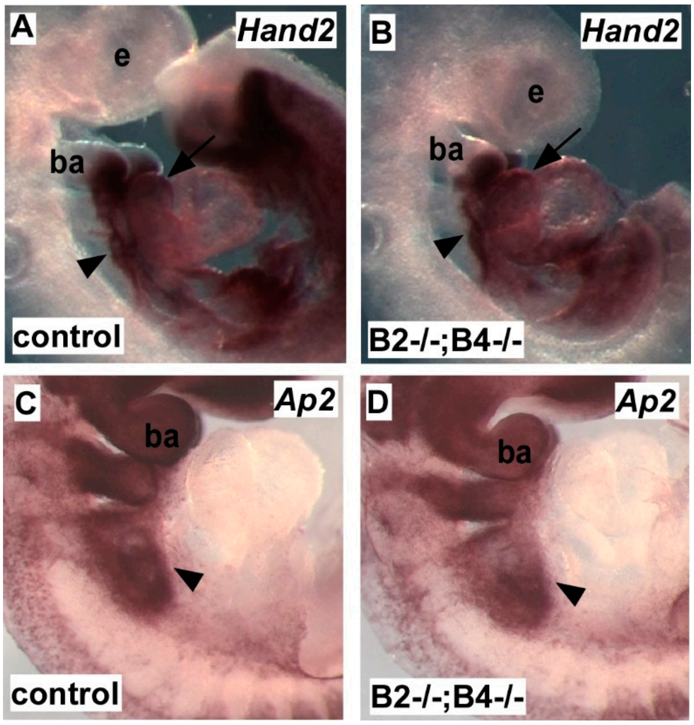

Figure S1. *Hand2* is not changed in control and *Bmp2/4* mutant. (**A, B**) Whole-mount in situ hybridization at E9.5 embryo showing *Hand2* expression (arrows and arrow heads). (**C, D**) Whole-mount in situ hybridization at E9.5 embryo showing *Ap2* expression (arrow heads). e, eye; ba, branchial arch.

: GCCG core consensus in GC-rich sequences
: Smad4 recognized Smad binding element (SBE) motif
: BMP-responsive element (BRE)

location on Chromosome 11 (Ensembl)

```
11:57647580 TAAGGTGGAAGACATCTGTAAGAACACCACGCCGGTGTTTGTGTGTGCCGGCGAAGGGAT
11:57647520 GAGCCTGTGTATATGTATGGACCTGTCATGGAGTCCAGGATGAAGACTGCTGGAGTCAGA
11:57647460 ACTGTGCTGTCTTGAGAATCCAGGCCCTTTCCACCCAGGTTCCCTCATTCTGAGAGTGAC
11:57647400 TCCCAACCCGCAGGGCACAAGAACCCGCGGAGCAGCTGCGCAGCTGCGGAACCGGCGCCT
11:57647340 TCCCGGGGAGCCTTCCCCAGGCCCCTGGCTGAGCCCAACCCCAGCGCTGCGGGCGCCAGG
11:57647280 CGCCTGCAAAAGCAGCGGGTTTCCAGTTCGCCTGGGGCAGGAAAGTGAGGTTCTCCGGAT
11:57647220 CTCTTTCCACTGCCGCTCACAATCTCTTGCACAACCAGTTCTCACAGGTGGGACCTTGGG
11:57647160 GCTCAGACCTCAGCGACTGAGCTTCCATCCACGCCTCAAAGCGAGATTACTCTCACAGAC
11:57647100 ACGCAGAAATCACAATTCGCACACATTCCTAGGATTTCACACACAGGGACTGTCATCTGT
11:57647040 TGAGCCCACGACCTACTCCACATTCGTTTTCACTTACCCACTTGCTACTGGGCCTCACAT
11:57646980 CCCGTCTGCGCACGGTGGCCTCTCTCCCACTGTCCCTGCCTGGTAATTGTCTTACACAAG
11:57646920 CTGGGCCTCAGCGAGCAACTTTCGAGTGGCTGGATCAGAAGCGAATTAAAAAGAATTTGA
11:57646860 CATCATCTCATTTCCCCCCTTAACTGACATTTGATAGGCACAAGCCCACCTAGGGAAGGA
11:57646800 GCGAGACCAGAAATGTTGGTGAGGATGGAGGGGGTCCTAGGGACAGGGACCTTAATGAAA
11:57646740 AGATGAAGAAGACCCAGAGTTTCAAGCTTAAAAGCCTGGTCTCCCACGTTCTTATCAACT
11:57646680 CTTGGAAAGCCATCTCTTTGTGGAATCCCACCTTGGAGAATCTAGGCTGATGGGGAGCAA
11:57646620 CACAAGGTTGCACAGATATTCCCTGCCTCAGCAGAGTGAGTGTGAAGCGCCTAGGCTGTT
11:57646560 TCTCCGTGGATATCAGGGGTGGGGGTGGGCTGGTTTCTTCCAGTGTGAGCCTCAGTGGAA
11:57646500 GTATCTTTATAGGATCAAAGATTTTCATGCAGGTGGCGAGCCAGATGGAGTGAGCAGCCC
11:57646440 AGCAGTCGCCGGCTCCTTAGCCCTACGTGCGAATCTTCCGAAGACACCGGGGAGTCCTGC
11:57646380 CACAGAGATGCCTTGGGGCCAAGGCGCCGCCAGGCTGCCTCGCCACCAGCTCAGCCTGCC
11:57646320 GGCGTTTACACCGAACCAGGCGCCTGAGAGGCTAGAGGAGCAGAGATCAGCCACCGTCTC
11:57646260 CACCCCCAACCCCCAAGCCCGGAACAAACTGGTCAGTGCCCAAGAGCTGGAAGCCCCGCT
```

Figure S2. Diagram of the Bmp/Smad regulatory element in the 5′ upstream of *Hand1* genomic sequence (at upper) and its phylogenetic sequence alignment (at lower).
